# Driving-related cognitive skills during antidepressant transcranial direct current stimulation: results in a subsample from the DepressionDC trial

**DOI:** 10.3389/fpsyt.2023.1255415

**Published:** 2023-12-07

**Authors:** Gerrit Burkhardt, Stephan Goerigk, Esther Dechantsreiter, Lucia Bulubas, Aldo Soldini, Peter Zwanzger, Julia Diemer, Frank Padberg, Alexander Brunnauer, Ulrike Kumpf

**Affiliations:** ^1^Department of Psychiatry and Psychotherapy, LMU University Hospital, LMU Munich, Munich, Germany; ^2^Charlotte Fresenius Hochschule, University of Psychology, Munich, Germany; ^3^kbo-Inn-Salzach-Klinikum, Clinical Center for Psychiatry, Psychotherapy, Psychosomatic Medicine, Geriatrics and Neurology, Wasserburg am Inn, Germany

**Keywords:** major depressive disorder, transcranial direct current stimulation, tDCS, depression, driving performance

## Abstract

Therapeutic transcranial direct current stimulation (tDCS) is a well-tolerated neuromodulatory intervention. However, there are currently no data on its impact on driving skills. Therefore, we conducted a validated assessment of driving-related cognitive skills in participants of the DepressionDC trial, a multicenter, randomized-controlled trial investigating the antidepressant effects of 6-week prefrontal tDCS in patients with major depressive disorder (MDD). Twenty-one patients (12 women, active tDCS, *n* = 11, sham, *n* = 10) underwent an assessment of driving-related cognitive skills before and after the intervention. Using a Bayesian analysis approach, we found no group differences between active tDCS and sham tDCS in the pre-post treatment changes for *visual perception* (estimated median difference: 3.41 [−3.17, 10.55 89%-CI], BF_01_: 2.1), *stress tolerance* (estimated median difference: 0.77 [−2.40, 4.15 89%-CI], BF_01_: 1.6), and *reaction time* (estimated median difference: 2.06 [−12.33, 16.83 89%-CI], BF_01_: 6.5). Our results indicate that repeated sessions of a conventional bifrontal tDCS protocol do not negatively impact driving-related cognitive skills in patients with MDD.

## Introduction

Transcranial direct current stimulation (tDCS) of prefrontal cortex regions is increasingly used as a neuromodulatory technique in various scientific and therapeutic applications (1). Conventional tDCS protocols are considered safe and well tolerated (1); however, there are currently no data on their long-term impact on driving skills. Driving is a context-dependent complex cognitive task (2) with high relevance for the daily functioning of many adults. Previous experimental studies have reported less risky driving behavior (3), improved car-following and lane-keeping (4), or no significant improvements in driving-related skills (5) after single tDCS sessions in healthy individuals. However, these results may not be generalizable to repeated tDCS sessions in patients with mental health disorders.

Therapeutic applications of tDCS, e.g., for the treatment of major depressive disorder (MDD), usually target the left dorsolateral prefrontal cortex (lDLPFC), a part of the frontoparietal network (FPN), with multiple treatment sessions across 2–6 weeks (6, 7). Since the FPN is implicated in the function of several cognitive domains like attention and working memory (8, 9), previous research has investigated whether such tDCS protocols elicit short-term effects on cognition. While a recent meta-analysis showed small effects of active tDCS versus sham tDCS on working memory and attention/vigilance across multiple neuropsychiatric disorders (10), another meta-analysis in patients with MDD reported no beneficial cognitive effects but reduced performance gains in processing speed (11). MDD has been associated with cognitive deficits, even following remission from a major depressive episode (12), and constitutes a potential risk factor for dementia (13). Correspondingly, patients with MDD also show impaired driving ability (14). Therefore, it is essential to rule out possible detrimental effects on driving-related cognitive skills and establish the road safety of new interventional methods used in this population.

We investigated the effects of a conventional bifrontal tDCS protocol on driving-related cognitive skills according to legal constraints with a standardized, computerized test battery in a subsample of participants from the recently published DepressionDC trial (7).

## Materials and methods

We recruited patients at two study sites (Munich and Wasserburg/Inn) of the recently published DepressionDC trial (Trial registration number: NCT02530164) (7) for an assessment of driving-related cognitive skills, which was optional for study participants. DepressionDC was a multicenter, randomized, sham-controlled trial investigating the efficacy of transcranial direct current stimulation (tDCS) in patients with MDD and no relevant psychiatric comorbidities in addition to a stable but not effective treatment with a selective serotonin reuptake inhibitor (SSRI). The trial comprised a 6-week acute treatment protocol with 2-mA bifrontal tDCS for 20 consecutive weekdays followed by two tDCS sessions a week for 2 weeks or sham treatments at the same intervals; each tDCS session lasted 30 min. Following the international electroencephalogram 10–20 system, two 35 mm^2^ sponge-covered rubber electrodes were placed over F3 (anode) and F4 (cathode). While active tDCS comprised a ramp-up phase before and a ramp-down phase after stimulation, sham tDCS consisted of ramp-up-ramp-down phases at the beginning and the end of each session to mimic the sensory artifacts of active stimulation. All treatment sessions were conducted at the respective study site. TDCS devices (DC-Stimulator Mobile, neuroConn, Ilmenau, Germany) were programmed to deliver active or sham tDCS based on a randomization code, without displaying any information on the treatment condition. The local ethics committees approved the study at each study site. All participants gave written informed consent before inclusion in the study.

We assessed participants’ driving-related cognitive skills at baseline and in the week after the last treatment session of the 6-week trial. Following the German guidelines for road and traffic safety (15), we applied a standardized, computerized psychomotor test battery comprising the following domains via a validated software^1^: (1) Visual perception was measured as the percentage of correct answers on the adaptive Tachistoscopic Traffic Perception Test (TAVT-MB). During the TAVT-MB, 20 images of typical traffic situations are presented to the test subject for 1 s each. After each image, subjects must respond to a 5-answer multiple-choice question on the contents of the displayed situation. (2) Reactive stress tolerance was measured as the number of omissions on the adaptive Vienna determination test (DT). In three test phases, subjects are presented with visual and acoustic stimuli to which they must respond by pressing several buttons, bars, and pedals using both their hands and feet. (3) Reaction time to simple stimulus constellations was measured as time in ms on the Choice-Reaction Test (RT), in which subjects must respond to a specific combination of visual and acoustic stimuli. Reaching at least a percentage above 15 is defined as a prerequisite to driving a car safely. The assessments lasted about 20–30 min for each participant.

All statistical analyses were conducted in R, version 4.2.1. We descriptively compared the global driving performance of participants in the active tDCS and sham groups using the Index of Psychomotor Performance (IPP) (16). The IPP is calculated by dividing the number of failed tests (participant falls short of the threshold of one standard deviation below the mean of normative data derived from a representative sample of car drivers in Germany) by the number of tests. Failure to more than 40% of tests is considered a severe impairment of driving skills. We then compared the mean changes from pre- to post-treatment between active tDCS and sham tDCS on the three domains using Bayesian linear regression (formula: change ~ treatment group) from the BayesFactor package (17), adjusting for mean centered baseline depression severity [assessed with the Montgomery–Åsberg Depression Rating Scale (MADRS)]. We chose a Bayesian approach to quantify the evidence in favor of the null hypothesis that changes in driving performance are similar between active tDCS and sham. 89%-credible intervals (CI) and Bayes Factors in favor of the null hypothesis (BF_01_) were computed using the bayestestR package (18). Interpretation of BF_01_ values followed Jeffreys (19).

## Results

**Figure 1:**
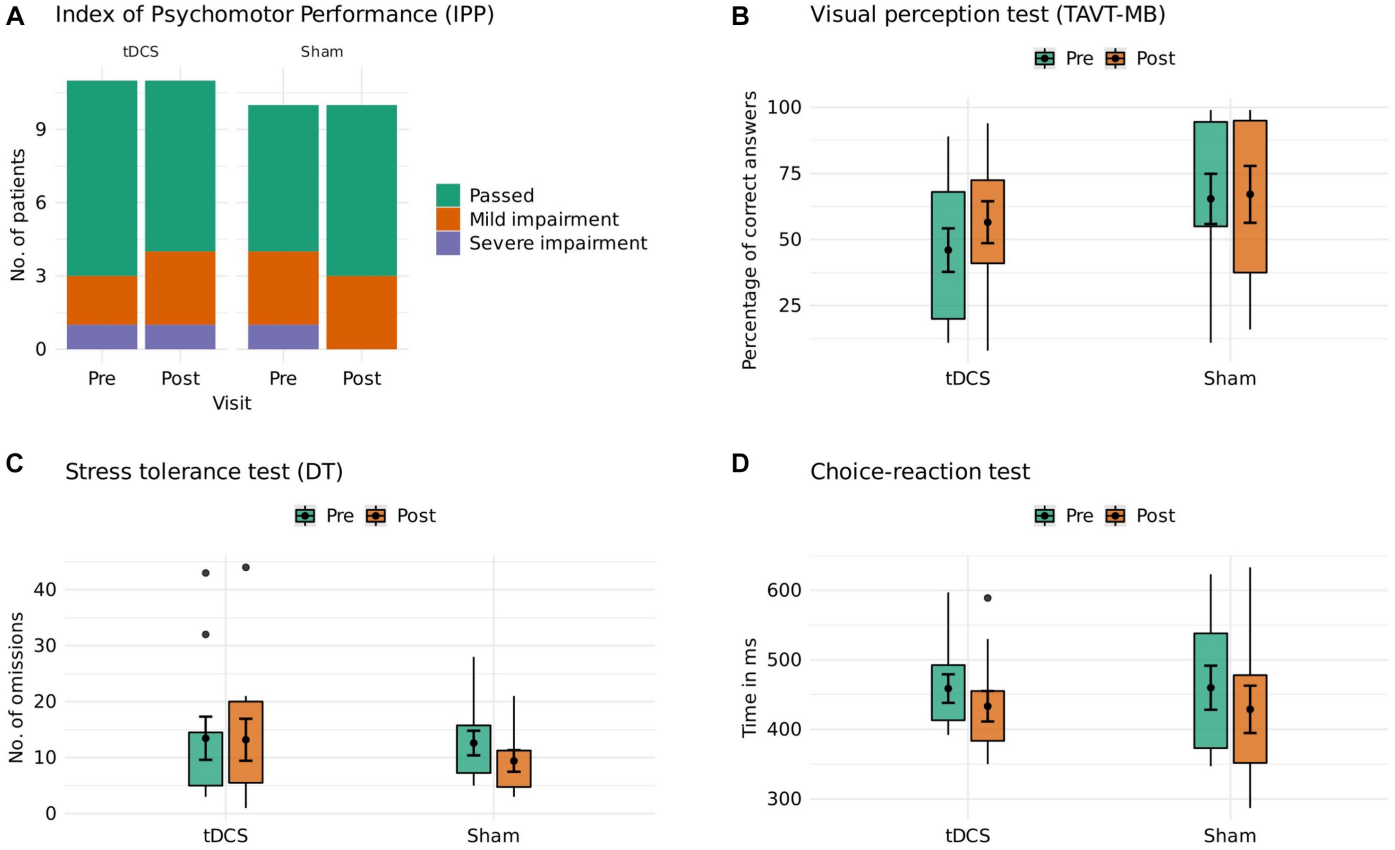
Driving-related cognitive skills.

Twenty-one patients (12 women, active tDCS, *n* = 11; sham, *n* = 10) underwent an assessment of their driving-related cognitive skills. The mean age in our sample was 39.1 years (SD 13.1). Further baseline characteristics are reported in Table 1. At baseline, 6 participants showed mild and 2 participants severe impairment of global driving skills. After the 6-week trial, one patient in the active tDCS group showed a relevant worsening (passed to mild impairment), and one patient in the sham group had a relevant improvement of global driving skills (severe impairment to passed). Comparisons of active tDCS and sham indicated anecdotal evidence against group differences for *visual perception* (estimated median difference: 3.41 [−3.17, 10.55 89%-CI], BF_01_: 2.1) and *stress tolerance* (estimated median difference: 0.77 [−2.40, 4.15 89%-CI], BF_01_: 1.6), as well as moderate evidence against group differences for *reaction time* (estimated median difference: 2.06 [−12.33, 16.83 89%-CI], BF_01_: 6.5). Group differences are visualized in Figure 1 and reported in Table 2. Single participant data are reported in Supplementary Table S1 and Supplementary Figure S1. A post-hoc sensitivity analysis adjusted for baseline duration of MDD episode did not change the overall results (Supplementary Table S2).

**Table 1:** Baseline characteristics of patients in the active tDCS and sham groups.

Characteristic	tDCS, *N* = 11	Sham, *N* = 10
Sex		
Female	8 (73%)	4 (40%)
Age—years	41 (13)	43 (15)
Age of depression onset—years	36 (12)	42 (14)
Duration of episode—weeks	33 (34)	53 (37)
SSRI		
Citalopram	2 (18%)	0 (0%)
Escitalopram	4 (36%)	7 (70%)
Fluoxetin	1 (9.1%)	0 (0%)
Paroxetin	1 (9.1%)	0 (0%)
Sertralin	3 (27%)	3 (30%)
MADRS at baseline	22.7 (7.7)	21.6 (5.2)
MADRS change at week 6	-6 (9)	−9 (8)

Mean (SD); n (%).

**Table 2:** Driving-related cognitive skills results.

Characteristic	tDCS, *N* = 11			Sham, *N* = 10			Between-group comparison of pre-post change		
	Baseline	Week 6	Pre-post change	Baseline	Week 6	Pre-post change	Baseline-adjusted median difference	89% CI	BF_01_
IPP class									
Passed	8 (73%)	7 (64%)	–	6 (60%)	7 (70%)	–	–	–	–
Mild impairment	2 (18%)	3 (27%)	–	3 (30%)	3 (30%)	–	–	–	–
Severe impairment	1 (9.1%)	1 (9.1%)	–	1 (10%)	0 (0%)	–	–	–	–
Visual perception test —correct answers	43 (20, 68)	67 (41, 72)	16 (−2, 24)	65 (30)	81 (38, 95)	0 (0, 5)	3.41	[−3.17, 10.55]	2.1
Stress tolerance test—no. of omissions	7 (5, 14)	8 (6, 20)	−2 (−7, 0)	12.0 (7.2, 15.8)	8.5 (4.8, 11.2)	−4 (−6, 0)	0.77	[−2.40, 4.15]	1.6
Choice-reaction task—time in ms	428 (413, 492)	414 (384, 455)	−19 (−58, −1)	446 (373, 538)	418 (352, 478)	−20 (−71, −6)	2.06	[−12.33, 16.83]	6.5

Median (ICR); n (%). 89%-CI, 89%-Credible Interval; BF_01_, Bayes Factor in support of the null hypothesis; IPP, Index of Psychomotor Performance.

## Discussion

Our results indicate that repeated sessions of a conventional bifrontal tDCS protocol do not negatively impact driving-related cognitive skills in patients with MDD. These results were consistent across three relevant standardized psychomotor test battery domains. All participants were on a stable dose of SSRI medication, which was continued during the tDCS trial. Thus, findings are unlikely to be confounded by pharmacological treatment effects. Furthermore, we controlled our analysis for depression severity to exclude potential effects of psychopathology on task performance.

Since patients in our study were aware they were participating in a driving skill assessment, the applied measures of visual perception, stress tolerance, and reaction time were context-dependent and might not have detected general cognitive effects of tDCS in these domains. For example, in contrast to a prior meta-analysis reporting a significant decrease in reaction time following tDCS stimulation of the DLPFC (20), our data showed moderate evidence against a group difference on this measure. Given that previously reported cognitive effects of tDCS were generally small (10, 20), future research should aim to investigate their real-world impact.

Our cohort consisted of patients with a mean age of 39 (SD 13.1) years, representing a typical MDD cohort. While our sample was too small to apply meaningful subgroup analyses, single-participant data showed that most patients with global baseline driving impairment were 55 and older, with heterogeneous performance trends across the study. MDD shows a significant overlap with mild cognitive impairment and manifest neurodegenerative disorders in older age groups (13, 21, 22). For DepressionDC, we excluded patients with relevant manifest comorbidities like dementia but did not apply more fine-grained assessments of prodromal or subthreshold cognitive and neurological impairments. Thus, our results indicate that a more specific focus on an older population is needed to ensure the road safety but also identify potential pro-cognitive effects of tDCS interventions in this age group.

Our study has several limitations. First, given the small sample size, these results may not be robust and should be considered as preliminary evidence. Second, compared to the overall trial sample, we recruited participants from the active tDCS group with lower MADRS change at week 6 (−6 vs. −8 points). This selection of participants with worse antidepressant response might have masked beneficial effects of tDCS on driving-related cognitive skills. However, this would not change our results in regards to driving safety. Third, our sample reached comparable high global driving skills at both time points; thus, the results might not be generalizable to more severely affected patient groups, like patients with schizophrenia (23). Last, we have not directly observed real-life driving behavior but used a validated test battery that has been shown to identify poor driving-related cognitive skills correctly.

In conclusion, we provide first evidence supporting the road safety of a conventional repeated tDCS protocol in patients with MDD. Further trials are needed that systematically assess the effects of non-invasive brain stimulation protocols (e.g., tDCS, but also repetitive transcranial magnetic stimulation) on driving-related cognitive skills in clinical samples as additional safety assessment.

## Data availability statement

The raw data supporting the conclusions of this article will be made available by the authors, without undue reservation.

## Ethics statement

The studies involving humans were approved by the Ethikkommission der Medizinischen Fakultät, Ludwig-Maximilians-Universität München. The studies were conducted in accordance with the local legislation and institutional requirements. The participants provided their written informed consent to participate in this study.

## Author contributions

GB: Conceptualization, Data curation, Formal analysis, Methodology, Project administration, Visualization, Writing – original draft, Writing – review & editing. SG: Conceptualization, Data curation, Methodology, Writing – review & editing. ED: Writing – review & editing. LB: Writing – review & editing. AS: Writing – review & editing. PZ: Writing – review & editing. JD: Conceptualization, Methodology, Writing – review & editing. FP: Conceptualization, Supervision, Writing – review & editing. AB: Conceptualization, Data curation, Methodology, Supervision, Writing – review & editing. UK: Conceptualization, Supervision, Writing – review & editing.

## Footnotes

^1^ https://www.schuhfried.com/vienna-test-system/
