# Supplementary material for: Driving-related cognitive skills during antidepressant transcranial direct current stimulation: results in a subsample from the DepressionDC trial
Source: Front Psychiatry. 2023 Dec 7;14:1255415. doi: 10.3389/fpsyt.2023.1255415 (PMC10733487; doi:10.3389/fpsyt.2023.1255415)

# Supplementary results

#

[Supplementary results 0](#_Toc150161526)

[Supplementary Table S1: Single participant data 1](#_Toc150161527)

[Supplementary Table S2. Driving-related cognitive skills results adjusted for MDD episode duration 2](#_Toc150161528)

[Supplementary Figure S1: Individual task performance 3](#_Toc150161529)

#

#

#

## Supplementary Table S1: Single participant data

| **Group** | **Age^1^** | **Sex** | **MADRS baseline** | **MADRS change** | **RT baseline** | **RT change** | **ATAVT baseline** | **ATAVT change** | **DT baseline** | **DT change** | **IPP baseline** | **IPP change** |
| --- | --- | --- | --- | --- | --- | --- | --- | --- | --- | --- | --- | --- |
| Sham | 55 | female | 22 | -6 | 580 | 53 | 64 | 5 | 17 | 4 | Mild impairment | Mild impairment |
| Sham | 26 | male | 16 | -15 | 385 | -98 | 99 | 0 | 10 | -6 | Passed | Passed |
| Sham | 58 | female | 26 | -5 | 623 | -143 | 29 | -12 | 16 | -8 | Mild impairment | Passed |
| Sham | 53 | male | 20 | -11 | 360 | -3 | 54 | 26 | 14 | -5 | Passed | Passed |
| Sham | 25 | male | 19 | -17 | 347 | -15 | 99 | 0 | 8 | -5 | Passed | Passed |
| Sham | 30 | male | 23 | -3 | 369 | -19 | 81 | 1 | 7 | 2 | Passed | Passed |
| Sham | 25 | male | 32 | 7 | 408 | -36 | 58 | -31 | 28 | -10 | Mild impairment | Passed |
| Sham | 60 | female | 22 | -22 | 555 | -83 | 11 | 5 | 6 | 1 | Severe impairment | Mild impairment |
| Sham | 54 | female | 23 | -3 | 485 | -20 | 60 | 23 | 5 | -2 | Passed | Passed |
| Sham | 46 | male | 13 | -10 | 487 | 53 | 99 | 0 | 15 | -3 | Passed | Mild impairment |
| tDCS | 23 | female | 15 | -8 | 480 | -66 | 22 | 45 | 13 | -3 | Passed | Passed |
| tDCS | 33 | female | 29 | -6 | 505 | -34 | 67 | 16 | 5 | 1 | Passed | Passed |
| tDCS | 28 | male | 20 | -2 | 428 | -78 | 34 | 19 | 7 | 13 | Passed | Mild impairment |
| tDCS | 24 | female | 17 | 2 | 392 | -14 | 69 | -1 | 15 | -8 | Passed | Passed |
| tDCS | 35 | female | 19 | 2 | 408 | -19 | 70 | 6 | 43 | -22 | Mild impairment | Passed |
| tDCS | 45 | female | 36 | 8 | 428 | 10 | 89 | -20 | 6 | 38 | Passed | Mild impairment |
| tDCS | 62 | female | 16 | -6 | 560 | 29 | 11 | -3 | 32 | -12 | Severe impairment | Severe impairment |
| tDCS | 55 | male | 12 | -11 | 424 | -50 | 18 | 21 | 5 | 0 | Passed | Passed |
| tDCS | 44 | male | 28 | -8 | 405 | -12 | 43 | 51 | 14 | -6 | Passed | Passed |
| tDCS | 52 | female | 29 | -24 | 418 | 21 | 67 | -45 | 3 | -2 | Passed | Passed |
| tDCS | 50 | female | 29 | -12 | 597 | -67 | 16 | 27 | 5 | -2 | Mild impairment | Mild impairment |

^1^Age in years. Abbreviations: Sham = sham tDCS; tDCS = active tDCS; MADRS = Montgomery–Åsberg Depression Rating Scale; RT = Choice-reaction task - Time in ms; ATAVT = Visual perception test (TAVT-MB) – correct answers; DT = Stress tolerance test - No. of omissions; IPP = Index of Psychomotor Performance

## Supplementary Table S2. Driving-related cognitive skills results adjusted for MDD episode duration

| **Characteristic** | **Between-group comparison of pre-post change** | | |
| --- | --- | --- | --- |
|  | **Baseline-adjusted median difference** | **89% CI** | **BF_01_** |
| Visual perception test - Correct answers | 1.72 | [-4.76, 8.77] | 5.7 |
| Stress tolerance test - No. of omissions | 0.58 | [-2.99, 4.34] | 5.8 |
| Choice-reaction task - Time in ms | 1.91 | [-13.45, 18.01] | 6.2 |

Median (ICR); n (%). 89%-CI=89%-Credible Interval; BF_01_=Bayes Factor in support of the null hypothesis.

## Supplementary Figure S1: Individual task performance


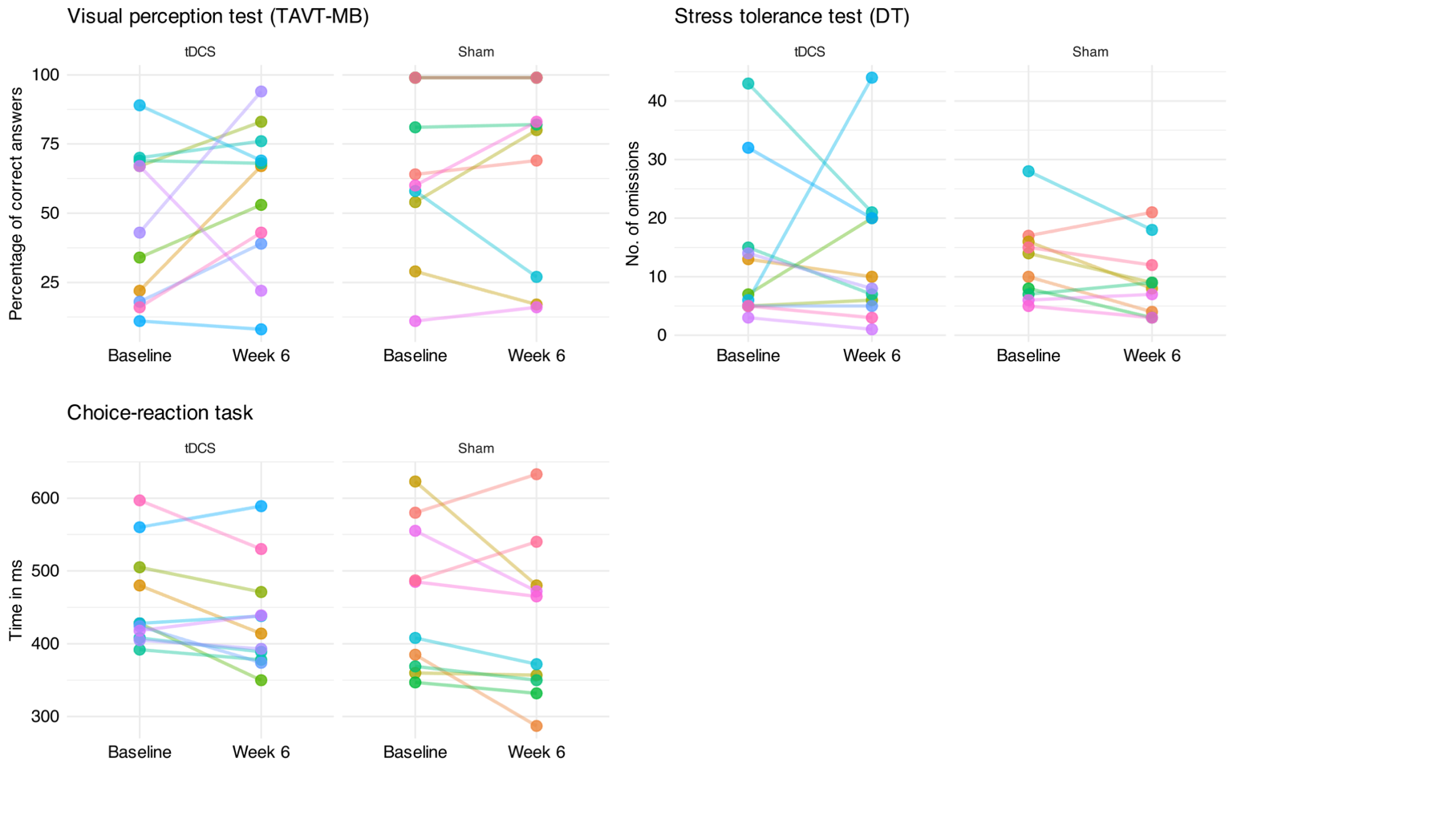

Supplement: Supplementary file 1 [file Data_Sheet_1.DOCX]
